# A Retrospective Case Series of Synovial Sarcoma of the Upper Extremity

**DOI:** 10.1155/2019/8704936

**Published:** 2019-08-01

**Authors:** J. Post, M. Houdek, A. L. Folpe, S. K. Kakar, B. K. Wilke

**Affiliations:** ^1^Beacon Memorial Hospital, South Bend, IN, USA; ^2^Mayo Clinic, Rochester, MN, USA; ^3^Mayo Clinic, Jacksonville, FL, USA

## Abstract

**Purpose:**

Previous studies have grouped the treatment of axial and appendicular synovial sarcomas. The purpose of this study was to assess the prognostic variables of upper extremity synovial sarcomas (UESS) and compare the outcomes of those who underwent a nononcologic or inadvertent excision prior to definitive resection to those who underwent an initial oncologic resection.

**Methods:**

We reviewed the records of 23 UESS treated with definitive surgery at our institution between 1990 and 2014. There were 13 women and 10 men with a median age of 30 years (6–60) and median follow-up of 63 months (15–248). Prognostic variables, recurrence-free survival (RFS), and overall survival (OS) were then assessed.

**Results:**

Fifteen patients (65%) had a prior unplanned excision. Five patients required an amputation to obtain local control of disease. There were 3 observed local recurrences and 2 distant metastases at a median of 45 months from presentation. We found no difference in need for amputation, RFS, or OS between those who had undergone a planned excision and those who had an unplanned excision.

**Conclusion:**

While we were unable to find a significant difference in outcomes or amputation rates between those who underwent reexcision of a previously unplanned excision and those who underwent an initial planned resection, the high rate of unplanned excision is troubling and should remind practitioners to consider sarcoma in the differential of all upper extremity masses.

## 1. Introduction

Soft tissue sarcomas of the upper extremity are a rare, heterogeneous group of malignancies. A 2003 epidemiology assessment of upper extremity tumors over a 25 year period found an annual incidence of approximately 2.2 soft tissue sarcomas per million persons in the United States [1]. Undifferentiated pleomorphic sarcoma (UPS) (formerly termed malignant fibrous histiocytoma (MFH)) has been the most common subtype reported in the largest upper extremity series, but age, race, and anatomic location have all been correlated to subtype incidence [1, 2]. Synovial sarcoma is a high-grade soft tissue sarcoma with significant metastatic potential, with main histologic subtypes including monophasic and biphasic types. Despite its name, it is generally a deep-seated extra-articular sarcoma occurring in the extremities of young adult patients [3]. Over 90% are characterized by a t (X; 18) (p11; q11) chromosomal translocation which produces the SS18-SSX1 or SS18-SSX2 fusion genes that can be detected by various cytogenetic or molecular genetic techniques, increasing the diagnostic accuracy [4].

Previous studies have attempted to identify prognostic variables associated with clinical outcomes in synovial sarcoma. Age [5–10], location [7, 11–14], size [8, 9, 11, 13–16], clinical stage [6, 8, 13, 15], histologic subtype [6, 7, 11, 17], fusion type [18–21], French Federation of Cancer Centers (FNCLCC) grade [22] surgical margin obtained [8, 12], and use of radiotherapy [6] have been previously reported as having an effect on prognosis but with conflicting results. Similar to other sarcomas, tumor stage and size have been identified as the most important prognostic factors of synovial sarcoma [18]. Metastatic progression of disease has been reported in 38% to 48% of cases, with the lung and regional lymph basins as the most common locations [7, 15, 17]. Modern multimodal (combination of surgery, radiation, or chemotherapy) treatment regimens report 5-year overall survivorship between 10% and 76%, with worse outcomes in patients presenting with metastatic disease [5–9, 15, 19, 21].

Given the rarity of synovial sarcoma, most previous studies have grouped all anatomic sites or combined both upper and lower extremity sarcomas. Better outcomes of hand and upper extremity sarcomas compared to axial locations have been reported [23], as has improved prognosis for small (less than 1 centimeter) hand and foot synovial sarcomas [16]. This may be in part related to the fascial compartments within the upper extremity that may limit tumor spread. Similarly, worse outcomes and more extensive salvage surgery have been reported with unplanned excisions of axial and appendicular synovial sarcomas performed at a nonspecialized facility [8, 13, 24]. The aim of this study is to examine synovial sarcomas isolated to the upper extremity to identify recurrence-free survival (RFS), overall survival (OS), and the associated prognostic variables. Specifically, we sought to compare the outcomes of primarily excised upper extremity synovial sarcoma with those who had had an unplanned or nononcologic surgery.

## 2. Materials and Methods

Following the institutional review board approval, we retrospectively reviewed the records of 328 patients with primary upper extremity sarcomas from our tumor database between 1990 and 2014. Thirty-six upper extremity synovial sarcomas were identified. We included patients who had definitive surgery at our institution and had a minimum of 12-month follow-up. We excluded those patients whose pathology was only reviewed at our institution but received definitive surgery/adjuvant treatments elsewhere or who had less than 12-month follow-up, leaving us with 23 patients for analysis.

All surgeries were performed by fellowship-trained orthopedic oncology and/or hand surgeons experienced in upper extremity sarcoma treatment principles. We defined a wide margin of excision in the upper extremity, based on the principles described by Enneking, as an en bloc resection with an anatomic barrier of uninvolved tissue such as fascia, paratenon, or periosteum outside the reactive zone. A marginal resection was defined as an excision through the reactive or inflammatory zone of the tumor [24]. Unplanned surgeries were defined as intralesional or excisional biopsies that were performed in a nononcologic manner (where no margin of normal tissue was taken around the tumor) [25]. Planned, definitive resections were those that had had adequate local imaging and were resected with a border of normal tissue defined previously as wide or had undergone core needle biopsy to obtain a definitive histopathologic diagnosis prior to resection. Upper extremity location was defined as shoulder girdle to finger-tip. Based on the previous work, 5 cm was used as a size threshold to differentiate large from small tumors [6, 11]. Maximal tumor size was recorded. Superficial and deep locations were defined with respect to the level of the investing fascia. Tumors were graded using the French Federation of Cancer Centers (FNCLCC) grading system; Grades 2 and 3 tumors were considered “high grade” for the purposes of this study [22].

**Figure 1:**
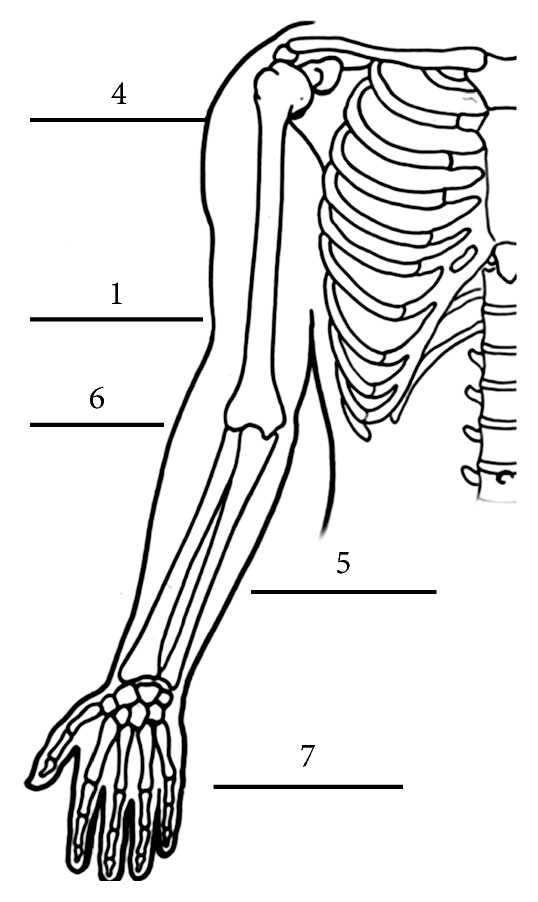
Graphical depiction of the incidence of upper extremity tumor locations by region.

All patients underwent magnetic resonance cross-sectional imaging (MRI) of the affected site and staging with chest computed tomography (CT). Positron emission tomography (PET) and sentinel lymph node biopsy (SLNP) were utilized selectively based on clinical nodal examination and risk stratification. The American Joint Committee on Cancer (AJCC) staging system was used to stage all patients when sufficient data were available [26]. There were 13 females and 10 males in our cohort with a median age of 30 years (range 6–60 years). The most common location was the hand and wrist (7 patients, 30%) followed by the forearm (5 patients, 22%) (Figure 1).

Resection for cure was the primary surgical goal, and negative margins were obtained in all primary cases as well as in reexcisions in those patients who had an inadequate initial excision. Median follow-up was 63 months (range 15–248). Local, regional, and distant recurrences were defined as either objective clinical, radiographic, or histologic findings as determined by the treating physician. Patients were followed with the clinical exam, contrast-enhanced magnetic resonance imaging, and chest computed tomography every 4 months for the first two years, every 6 months from years 2–5, and annually thereafter for surveillance. All available histologic specimens (18 of 23 cases) were re-reviewed and confirmed as synovial sarcoma by a musculoskeletal pathologist (ALF) using published diagnostic criteria [4].

Continuous variables were compared using unpaired Student's *T*-tests, and categorical variables were compared with the Fisher Exact tests. The Kaplan–Meier survival method was used to estimate overall and recurrence-free survival. Proportional hazard regression analysis was performed to assess the association of covariates with the risk of recurrence. All calculations were made with statistical significance set at a *p* value < 0.05.

## 3. Results

Clinicopathologic demographics can be found in Tables 1 and 2. There were 8 patients who underwent planned excision at our institution and 15 patients who had had a nononcologic resection or unplanned surgery prior to definitive reexcision at our institution. We found no differences in patient age, sex, tumor depth, tumor volume, presence of metastases at presentation, or the need for amputation to obtain local control between groups. All tumors were high grade, and thirteen patients (57%) demonstrated monophasic histology (demonstrating only spindle cells). Fifteen (65%) were deep to the fascia. Only four tumors were >5 cm. There was one case of osseous invasion of the metacarpal and no cases of vascular invasion. Fourteen patients (61%) were AJCC stage II at presentation (high grade, tumor size <5 cm, no nodal, or distant metastases).

After risk stratification by a multispecialty team comprised of medical/radiation/orthopedic oncologists, eight patients received neoadjuvant chemotherapy (35%). Doxorubicin and ifosfamide were the most common regimens utilized. Treatment regimens were based on age, the ability to tolerate the medications, size of tumor, and presenting stage. Seventy-eight percent of patients received multimodal treatment with a combination of neoadjuvant, intraoperative, and adjuvant chemotherapies and irradiation. Sixty-five percent (15/23) of patients underwent neoadjuvant external beam radiation with a median dose of 50 Gy (45–60 Gy). All patients underwent surgical resection with curative intent, and negative margins were obtained in all patients. To obtain local control and negative surgical margins, there were 3 patients in the unplanned group and 2 in the planned group who required amputation of a digit or limb. Six patients (23%) underwent intraoperative radiation (median 1,000 cGy), and four patients were treated with additional brachytherapy (median 1,500 cGy). The decision to employ these intraoperative adjuvants was made by the treating surgeon and radiation oncologist.

In our cohort, we did not find RFS to be affected by the previous unplanned surgery (*p*=0.46). Similarly, RFS was not found to be affected by age, neoadjuvant therapies, intraoperative radiation, brachytherapy, tumor depth, tumor size, wide resection, or histologic subtype (*p* > 0.05). Administration of postoperative chemotherapy was the only factor found to be associated with recurrence-free survival (*p*=0.002) (Table 3).

**Figure 2:**
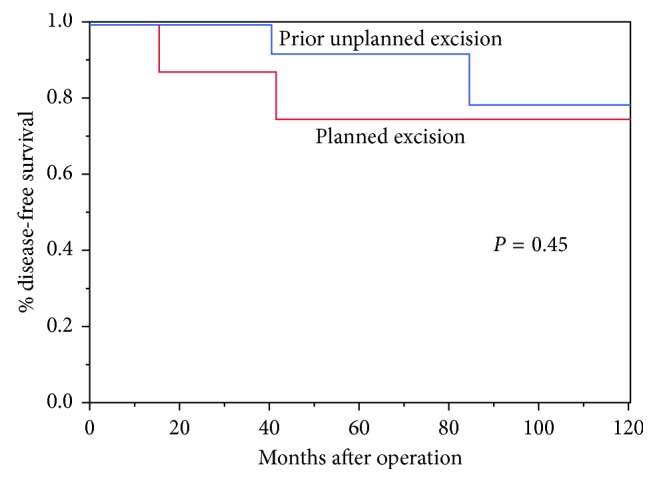
Kaplan–Meier analysis demonstrating no observed difference (*p*=0.45) in recurrence-free survival between planned and unplanned excisions.

One patient in each subgroup went on to develop pulmonary metastases following surgical resection. One additional patient in the planned resection cohort presented with metastatic disease. The patient in the unplanned excision group developed pulmonary metastases at 40 months after resection. He underwent metastasectomy and is alive with no evidence of disease at 17.5 years. The other patients with metastases died of their disease at a mean of 6 months. Two patients (9%) died of disease. Local recurrence was observed in 3 patients (13%). The mean time from surgical resection until recurrence was 45 months (15–84). We found no difference in 5- and 10-year RFS between groups (*p*=0.45). The RFS for unplanned excisions was 92% at 5 years and 79% at 10 years compared to 75% at both 5 and 10 years for planned surgeries (Figure 2). Although not reaching statistical significance, metastases at presentation and distal recurrence (metastases) correlated with a worse overall survival (Table 4). Overall survival for all patients was 91% at both 5 and 10 years.

There were six patients (23%) with reported treatment complications. Three soft tissue contractures developed requiring surgical release and flexor tenosynovectomy (two thenar web spaces and one carpal tunnel). There were two infections requiring further operative debridement and two painful neuroma formations requiring neurectomy (common digital nerve and superficial sensory branch of the radial nerve).

## 4. Discussion

Previous studies have examined prognostic variables associated with synovial sarcoma, but most of these studies have grouped all anatomic sites together [5–9, 11, 12, 15–20]. Similarly, previous studies have looked at the effect of unplanned excisions of soft tissue sarcoma on prognosis, but most of these studies have grouped low- and high-grade tumors with heterogeneous subtypes and locations [25–31]. The median age of our upper extremity series was 30 years (6–60) which is consistent with previous large series which found the incidence highest in the 4^th^ decade [6, 7, 9, 11, 13].

Despite the varied prognostic factors previously described [5–17], most series have found that young patients with <5 cm lesions in the extremities have better long-term survival than large, axial-based tumors in older individuals [4, 5, 9, 10]. These factors have led some to create multimodal treatment arms utilizing surgical excision, chemotherapies, and irradiation based on preoperative nomograms [14, 32]. In our series, eight patients received neoadjuvant chemotherapy. Doxorubicin and ifosfamide were the most common regimens utilized. Postoperative chemotherapy was found to statistically affect recurrent-free survival (*p*=0.002), but this was likely a result of selection bias in high-risk patients.

We observed an overall local recurrence rate of 13% at a median of 63 months, similar to studies by Choong et al. [8] and Lewis et al. [15], which demonstrated a 10 and 12% 5-year recurrence rates, respectively, but substantially lower than the 30% local recurrence rate observed by Ferrari et al. [9]. Two local recurrences occurred in the planned excision subgroup and one in the reexcision group. While we observed a trend towards a lower risk of local recurrence in the unplanned excision group, this was not statistically significant and likely is a reflection of the more superficial location of these tumors compared to the planned resection cohort. Finally, pulmonary metastases at presentation were low (4%) which is similar to the 6% observed in the largest single institution series of 271 patients by Ferrari et al. [9].

The literature supports surgical reexcision following unplanned surgery for extremity soft tissue sarcoma [27–31]. A large series of 407 reexcisions for unplanned extremity soft tissue sarcomas from Memorial Sloan Kettering was compared to a cohort of patients undergoing primary excision. Even after controlling for size, stage, and margin status obtained, the authors found a survival benefit with reexcision and concluded that the liberal reexcision of soft tissue sarcomas was indicated [28]. One of the limitations of these excision series is their heterogeneous grouping of soft tissue sarcomas. Low- and high-grade tumors of various subtypes and axial and appendicular locations are often aggregated together, limiting definitive prognostic conclusions. Synovial sarcomas only accounted for 5–17% of the reexcised subtypes in several previous large series [27, 29, 30].

A strength of our study was that the cases were identified through a reliable upper extremity sarcoma database at a large tertiary referral center. An expert in musculoskeletal sarcoma pathology re-reviewed and confirmed all available histopathologic specimens. Several limitations, however, should be acknowledged. Our study is limited by a small cohort and is almost certainly underpowered. Some data, including original sizes of tumors and histopathologic samples, were not available for re-review. Due to the high number of previously excised tumors, histologic grading was not practical on all specimens as no residual sarcoma was identified in 5 reexcisions and original outside histopathology slides were not available for re-review. Although the median follow-up was 63 months, distant recurrence has been described to occur late with some authors recommending 10-year postresection surveillance [13]. Treatment protocols were also not standardized and were dynamic over the review period and subject to selection bias. Functional outcomes of limb salvage versus amputation and planned versus reexcision of unplanned surgeries were not examined.

## 5. Conclusion

The majority of upper extremity synovial sarcomas presented following an unplanned excision at an outside facility. We were unable to find a significant difference between the need for amputation between groups. The majority of upper extremity synovial sarcomas were smaller than 5 cm and presented at an early clinical stage. Pulmonary metastases were rare and were associated with a poor prognosis. We were unable to find a significant difference between recurrence-free survival and overall survival between patients undergoing planned and unplanned excisions. Wide reexcision of previously unplanned excisions of upper extremity synovial sarcoma was associated with a low rate of local recurrence and similar recurrence-free and overall survival compared to patients who underwent primary planned excisions. These findings can help counsel upper extremity patients diagnosed with synovial sarcoma, particularly those who have had an unplanned excision.

## Figures and Tables

**Table 1:** Clinicopathologic demographics of patients who underwent an unplanned excision of an upper extremity synovial sarcoma.

Case	Age (years)	Sex	Location	Size	Depth	Stage	Surgical treatment	Adjuvant treatments	Local recurrence	Mets	Follow-up (months)	Outcome
1	30	Male	Elbow	<5 cm	Deep	IIA	Wide reexcision	Brachytherapy AXRT	No	No	155	ANED
2	40	Female	Thenar eminence	<5 cm	Superficial	IIA	Wide reexcision FDS/FDP/ABP resection Tendon transfers	Chemo NXRT IORT	No	No	248	ANED
3	57	Male	Index finger	Unknown	Deep	Unknown	Ray resection index/long fingers	None	No	No	16	ANED
4	55	Female	Brachial plexus	Unknown	Deep	IIA	Wide reresection, pectoralis flap	NXRT IORT	No	No	161	ANED
5	22	Female	Ring finger	<5 cm	Superficial	IIA	Ray resection ring finger	None	No	No	95	ANED
6	19	Female	Forearm	<5 cm	Deep	IIA	Wide reexcision	NXRT IORT	No	No	92	ANED
7	39	Male	Antecubital fossa	<5 cm	Deep	IIA	Marginal reexcision Radial forearm flap	Chemo NXRT brachytherapy	No	Lung	210	ADP
8	13	Female	Trapezius	>5 cm	Deep	III	Wide reexcision	IORT AXRT	No	No	84	ANED
9	6	Female	Forearm	<5 cm	Deep	IIA	Wide reexcision	None	No	No	60	ANED
10	40	Male	Hand	<5 cm	Superficial	IIA	Marginal reexcision Radial forearm flap	NXRT	No	No	51	ANED
11	55	Female	Long finger	<5 cm	Superficial	IIA	Ray resection long finger	None	No	No	60	ANED
12	41	Male	Elbow	Unknown	Superficial	Unknown	Wide reexcision	NXRT	No	No	63	ANED
13	31	Female	Forearm	>5 cm	Deep	IIB	Wide reexcision Free serratus flap	Chemo NXRT	No	No	90	ANED
14	30	Male	Elbows	Unknown	Superficial	Unknown	Wide reexcision	Chemo NXRT	Yes	No	56	ANED
15	22	Female	Forearm	<5 cm	Superficial	IIA	Wide reexcision	NXRT	No	No	55	ANED

AXRT, adjuvant radiation; Mets, metastases; NXRT, neoadjuvant radiation; IORT, intraoperative radiation; chemo, chemotherapy; ANED, alive no evidence of disease; LTF, lost to follow-up; ADP, alive with disease progression.

**Table 2:** Clinicopathologic demographics of patients who underwent a planned excision of an upper extremity synovial sarcoma.

Case	Age (years)	Sex	Location	Size	Depth	Stage	Surgical treatment	Adjuvant treatments	Local recurrence	Mets	Follow-up (months)	Outcome
1	17	Female	Forearm	<5 cm	Deep	IIA	Wide excision	Chemo NXRT Brachytherapy	No	No	54	ANED
2	13	Female	Deltoid	>5 cm	Deep	IIB	Wide excision	Chemo NXRT	No	No	84	ANED
3	18	Male	Thenar webspace	<5 cm	Deep	IIA	Marginal excision	Chemo NXRT Brachytherapy	No	No	74	ANED
4	51	Male	Brachial plexus	<5 cm	Deep	IV	Wide excision	Chemo NXRT	No	Lung^*∗*^	9	DOD
5	60	Female	Antecubital fossa	<5 cm	Deep	IIA	Wide excision Sural nerve grafting Radial forearm flap	Chemo NXRT IORT	No	No	54	ANED
6	10	Female	Elbow	<5 cm	Deep	IIA	Wide excision MCL reconstruction radial forearm flap	None	No	No	59	ANED
7	19	Male	Index finger	>5 cm	Superficial	IIB	Ray resection	Chemo NXRT	Yes	No	15	ADP
8	45	Male	Proximal arm	Unknown	Deep	Unknown	Forequarter amputation	Chemo	Yes	Lung	3	DOD

^*∗*^On presentation; Mets, metastases; NXRT, neoadjuvant radiation; IORT, intraoperative radiation; chemo, chemotherapy; ANED, alive no evidence of disease; ADP, alive with disease progression; DOD, died of disease; MCL, medial collateral ligament.

**Table 3:** Factors affecting recurrence-free survival.

	Hazard ratio	*p* value
Age ≤30	1.41 (0.16–11.90)	0.72
Deep to fascia	0.54 (0.06–4.56)	0.54
Wide excision	0.74 (0.09–15.23)	0.80
Preoperative chemotherapy	0.67 (0.03–5.30)	0.72
Postoperative chemotherapy	23.77 (3.03–481.39)	0.002
Brachytherapy	1.46 (0.07–11.57)	0.74
Postoperative radiation	1.67 (0.08–13.23)	0.67
Unplanned excision	0.47 (0.05–4.03)	0.46
Size >5 cm	4.67 (0.55–39.18)	0.14
Mass distal to elbow	0.32 (0.01–2.55)	0.29

**Table 4:** Factors affecting overall survival.

	Hazard ratio	*p* value
Monophasic pathology	0.61 (0.02–11.90)	0.73
Metastases at presentation	21.97 (0.86–555.50)	0.06
Preoperative chemotherapy	1.70 (0.06–43.15)	0.70
Local recurrence	8.36 (0.32–212.93)	0.16
Distant recurrence	15.19 (0.58–393.33)	0.08
Any recurrence	5.62 (0.22–142.62)	0.24
Need for amputation	4.13 (0.16–104.64)	0.33

## Data Availability

The data used to support the findings of this study are available from the corresponding author upon request.

## Additional Points

The investigation was performed at the Mayo Clinic in Rochester, MN.
